# Biosorption of Arsenic(III) from Aqueous Solutions by Modified Fungal Biomass of *Paecilomyces* sp.

**DOI:** 10.1155/2013/376780

**Published:** 2013-10-23

**Authors:** Ismael Acosta Rodríguez, Víctor M. Martínez-Juárez, Juan F. Cárdenas-González, María de Guadalupe Moctezuma-Zárate

**Affiliations:** ^1^Universidad Autónoma de San Luis Potosí, Facultad de Ciencias Químicas, Centro de Investigación y de Estudios de Posgrado, Laboratorio de Micología Experimental, Avendia Dr. Manuel Nava No. 6, Zona Universitaria, 78320 San Luis Potosí, SLP, Mexico; ^2^Área Académica de Medicina Veterinaria y Zootecnia, Instituto de Ciencias Agropecuarias, Universidad Autónoma del Estado de Hidalgo, Zona Universitaria, Rancho Universitario Km 1, 43600 Tulancingo de Bravo, HGO, Mexico

## Abstract

The biosorption of As(III) on iron-coated fungal biomass of *Paecilomyces* sp. was studied in this work. It was found that the biomass was very efficient removing the metal in solution, using Atomic Absorption, reaching the next percentage of removals: 64.5%. The highest adsorption was obtained at pH 6.0, at 30°C after 24 hours of incubation, with 1 mg/L of modified fungal biomass.

## 1. Introduction 

Arsenic, a common element in nature, is a naturally occurring contaminant of drinking water and can be found in the earth's crust, ground, and marine water and in the organic world as well. It is mobilized through a combination of natural processes such as weathering reactions, biological activity, and volcanic emissions [1, 2] as well as through a range of anthropogenic activities such as gold mining, nonferrous smelting, petroleum refining, combustion of fossil fuel in power plants, and the use of arsenical pesticides and herbicides [3, 4]. Contaminated groundwater by arsenic is a well-known environmental problem that can have severe human health implications. Chronic exposure to arsenic concentrations above 100 ppb can cause vascular disorders, such as dermal pigments (Blackfoot disease) and skin, liver, and lung cancer [5, 6]. An arsenic concentration of 10 *μ*g/L has been recommended by World Health Organization as a guideline value for drinking water [7].

Arsenic is found in soils and natural waters, mainly, in the form of arsenate [As(V)] and arsenite [As(III)]. The distribution between dissolved As(III) and As(V) is dependent on redox potential and pH. Under oxidizing conditions, the predominant specie is As(V), which exists as deprotonated oxyanions of arsenic acid (H_2_AsO^4−^, HAsO_4_^2−^, and AsO_4_^3−^). Under reducing conditions, As(III) is thermodynamically stable and exists in solution as arsenious acid, a neutral, uncharged molecule (H_3_AsO_3_^0^) that only forms deprotonated oxyanions at pH > 9.2 (H_2_AsO^3−^ and HAsO_3_^2−^) [8]. The As(III) species are more toxic than As(V). At the pH of most natural soils and water, As(III) is not strongly adsorbed on most mineral surfaces because is electrically neutral compared with the negatively charged of As(V) oxyanions [9]. 

Iron oxide-coated sand was used in many studies for arsenic removal, and the results were positives [10], and with modified [iron(III) loaded] orange juice industrial residue, [11], with *Penicillium purpurogenum* [12], enhancement in arsenate removal by chemically (polyelectrolyte, dodecylamine, and cetyltrimethylammonium bromide) modified *Penicillium chrysogenum* compared with the unmodified biomass [13], the biovolatilization of As by *Aspergillus clavatus*, *Aspergillus niger*, *Trichoderma viride*, and *Penicillium glabrum* [14], the biological removal of arsenic pollution by *Aspergillus* and *Trichoderma*, *Neocosmospora* sp., *Sordaria* sp., *Rhizopus* sp., *Penicillium* sp., and sterile mycelia strain fungal groups [15], and the modified fungal biomass of *A. niger *[16]. The objective of this work was to study the removal of Arsenic(III) in solution by the modified fungal biomass of *Paecilomyces* sp.

## 2. Experimental

### 2.1. Microorganism and Culture Conditions

A chromate resistant filamentous fungus was isolated from polluted air with industrial vapors, near of Chemical Science Faculty, located in the city of San Luis Potosí, Mexico, in Petri dishes containing modified Lee's minimal medium [LMM, 16] [with 0.25% KH_2_PO_4_, 0.20% MgSO_4_, 0.50% (NH_4_)_2_SO_4_, 0.50% NaCl, 0.25% glucose] supplemented with 500 mg/L K_2_CrO_4_; the pH of the medium was adjusted and maintained at 5.3 with 100 mMol/L citrate-phosphate buffer. The cultures were incubated at 28°C for 7 days. The strain was identified based on their morphological structures such as color, diameter of the mycelia, and microscopic observation of spores [17]. The fungus was grown at 28°C in agitation and aerated liquid media containing thioglycolate broth, 8 g/L. After 4-5 days of incubation, the cells were centrifuged at 3000 rpm for 10 min, washed twice with trideionized water, and then dried at 80°C for 4 h in oven. Finally, the fungal biomass was milled and stored in an amber bottle in the refrigerator until their use. 

### 2.2. Arsenic(III) Solutions

For analysis, a series of solutions of Arsenic(III) (NaAsO_2_) of 1 mg/L were prepared, pH was adjusted with nitric acid and/or NaOH, and the quantity of biomass added to each flask was of 1 g/100 mL for the arsenic's solution. It took samples at different times, the biomass is removed for centrifugation (3000 rpm/5 min), and the supernatant is analyzed to define the ion metal concentration.

### 2.3. Preparation of Iron Oxide-Coated Biomass

A total 80 mL of the 2 M Fe(NO_3_)_3_·9H_2_O was prepared, and 1.0 mL of 10 M NaOH was added to this solution and mixed thoroughly. A total 20 g of the fungal biomass powder was taken in a porcelain pot, a mixture of iron oxide and NaOH solution was added to the porcelain pot and homogenized and kept in an oven for 3 h, at 80°C. After 3 h the oven temperature was raised to 110°C and continued for 24 h. The coated biomass powder was separated by crushing with mortar and pestle [16].

### 2.4. Determination of Arsenic(III)

The concentration of arsenic ions in solution was determined spectrophotometrically by Atomic Absorption (Atomic Absorption Spectrometer Varian, Model Spectra AA-20). 

## 3. Results and Discussion

### 3.1. Arsenic Removal by Untreated and Iron Oxide-Coated Biomass at Different pH

**Figure 1:**
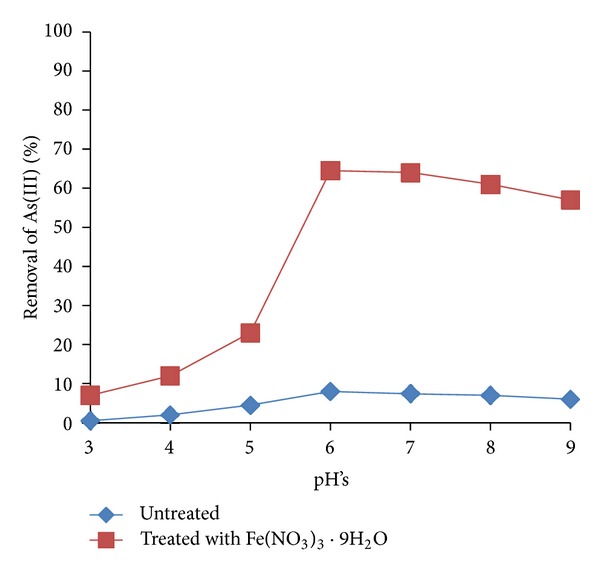
As(III) removal by untreated and treated iron-coated biomass at different pH's. 1 mg/L As(III), 30°C, 100 rpm, 1 g of fungal biomass of *Paecilomyces* sp.

Figure 1 shows the effect of untreated and iron-coated biomass and pH on biosorption of As(III) ions (1 mg/L, 24 h) to the dried *Paecilomyces *sp. biomass. It was found that the removal is very low in untreated biomass, because at 24 h of incubation and pH 7.0, there is an 8.4% (0.084 mg/L) of removal (Figure 1), and these results are better than those reported for *A. clavatus*, *A. niger*, *T. viride*, and *P. glabrum* [14], which were able to remove between 0.010 to 0.067 *μ*g/L, and they are lower than *Penicillium purpurogenum*, 3.4 (mg/g of biomass) [12], and stem of *Acacia nilotica*, 0.19 mg/L [18]. Structural properties of the biosorbent including the cellular support and other several factors are known to affect the biosorption rate [19]. With respect to an iron-coated biomass, the removal of As(III) is very efficient (64.5%, pH 6.0, 24 h) (Figure 1). As(III) is better adsorbed or greater than pH 6.0 because it is partially ionized in H_2_AsO^−^_3_ form at a pH of 6.0 or above 9.22 and form complexes with the iron oxide-coated biomass. At higher pH, OH^−^ ions in the solution increase compete with the arsenate ions, and the adsorption of As(III) is reduced [20]. Considering that there was no other chemical reaction between the iron oxide and the biomass, mechanism of As(III) removal could be similar to arsenic adsorption on iron oxide-coated sand [10]. These results are similar to the other reports: by iron(III)-poly (hydroxamic acid) complex [3], iron oxide-coated sand [10], chemically modified fungal biomass [13], iron oxide-coated biomass of *A. niger* [16], mixed metal oxide impregnated chitosan beads [20], and modified iron activated carbon [21].

### 3.2. The Effect of Incubation Time

**Figure 2:**
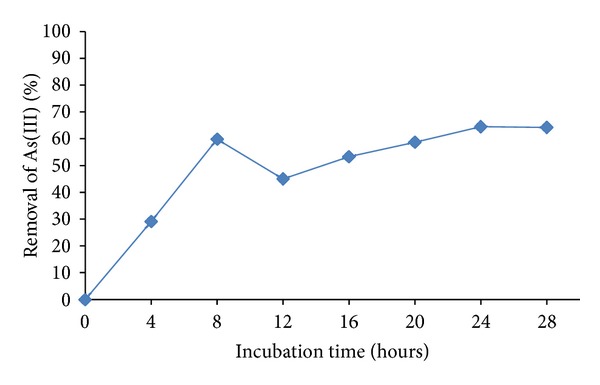
The effect of the Incubation time of As(III). 1 mg/L As(III), 30°C, 100 rpm, pH 6.0, 1 g of iron-coated fungal biomass of *Paecilomyces* sp.

The biosorption of As(III) onto iron-coated biomass, with different time (0–24 h) As(III), solutions of 1 mg/L, at pH 6.0, and 1 g/L of dosage of biosorbent material, is shown in Figure 2. The sorption study of As(III) ions onto biosorbent material as a function of contact time showed that sorption is optimum at 24 h, which indicates availability of the biosorption sites. The kinetics of sorbent metal interaction at optimum pH may be acknowledged to enhance accessibility of the chelating sites of the biosorbent material [22]. Further increase in time, no significant enhancements were observed in removal of As(III). These results are similar for modified iron activated carbon, with a removal of 1 mg/100 mL at 24 h [21], and are different to the others reports: iron oxide-coated biomass of *A. niger*, 80.1 mg/L, 12 h) [16], and with powder of stem of *Acacia nilotica*, (0.19 mg/L, 15 min, 4 g of biomass) [18].

### 3.3. Effect of Temperature

**Figure 3:**
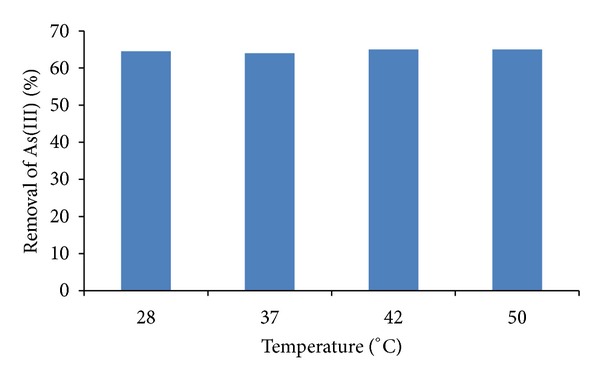
The effect of the temperature on As(III) removal. 1 mg/L As(III), 100 rpm, pH 6.0, 1 g of iron-coated fungal biomass of *Paecilomyces* sp.

Figure 3 shows the effect of different temperatures (30°C, 37°C, 42°C, and 50°C). The adsorption capacity was similar in all temperatures analyzed (64%-65%), and similar to the reported for iron oxide-coated biomass of *A. niger*, [16], and different of *A. nilotica *[18]. The temperature of the adsorption medium could be important for energy-dependent mechanisms in metal biosorption by microorganisms. Energy independent mechanisms are less likely to be affected by temperature since the process responsible for biosorption is largely physicochemical in nature. The biosorption of As(III) by *Paecilomyces* sp. fungus seems to be nondependent of the temperature tested (30–50°C). 

### 3.4. Effect of Initial As(III) Concentration

**Figure 4:**
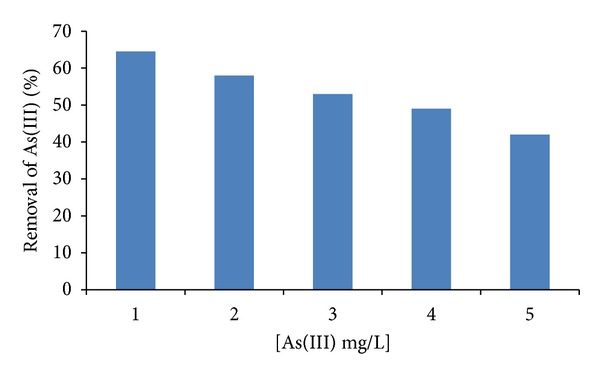
The effect of the concentration of As(III) in solution on the removal of As(III) ions. 100 rpm, 30°C, pH 6.0, 1 g of iron-coated fungal biomass of *Paecilomyces* sp.

Biosorption capacities of *the Paecilomyces* sp. iron-coated biomass for the As(III) ions were studied as a function of the initial As(III) ions concentration between 1 and 5 mg/L in the biosorption medium (Figure 4). The uptake of As(III) found to increase as the initial metal concentration is low. It was because the number of ions adsorbed from solutions of lower concentrations is more than that removed from high concentrated solutions. The uptake of As(III) was observed 64.5% and 58% at lower concentrations (1-2 mg/L) and 49% and 42% at higher concentrations (4-5 mg/L). A similar type of trend was reported for the removal of As(III) with stem of *A. nilotica* [18] and Hg(II) from aqueous solution by sorption on *R. oligosporus* [23]. Although a direct comparison between the examined treated biomass with those obtained in literature is difficult, due to the varying experimental conditions employed, *Paecilomyces* sp. iron-coated biomass showed reasonably high sorption efficiency as compared with other adsorbents. More specifically, green algae *Ulothrix cylindricum*, Fe(III)-treated biomass of *Staphylococcus Xylosus*, *Inonotus hispidus* biomass, and Al/Fe modified montmorillonite exhibited higher biosorption capacities of As(III) at 67.2, 54.35, 51.9, and 18.19 mg/g, respectively, [24–27], whereas other biosorbents including *Acidithiobacillus ferrooxidans* BY-3, *Bacillus* sp. strain DJ-1, agricultural residues, modified fungal biomass of *A. niger*, and iron oxide-coated sand exhibited lower values of maximum uptake capacity at 277.22 *μ*g/g, 6.14 *μ*g/g, 138.88 *μ*g/g, 75 *μ*g/100 mg, and 41.1 *μ*g/g, for As(III), respectively, [10, 16, 28–30]. Taking into consideration that *Paecilomyces* sp. has been previously used for the effective removal of Cr(VI) [31], the results of this study render this strain is very efficient adsorbents for the removal of toxic ions from aqueous environments.

### 3.5. Effect of Initial Biomass Concentration and Application on Natural Water

**Figure 5:**
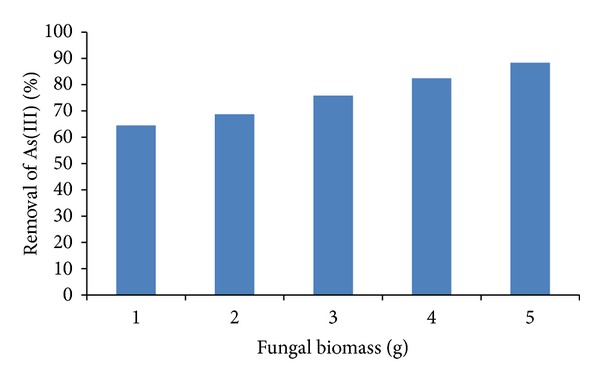
The effect of fungal biomass concentration on the removal of As(III). 1 mg/L As(III), 100 rpm, 30°C, pH 6.0.

The influence of biomass on the removal capacity of As(III) was depicted in Figure 5. If we increase the amount of biomass also increases the removal of the metal in solution (88.3% of removal, with 5 g of fungal biomass at 24 hours), with more biosorption sites of the same, because the amount of added biosorbent determines the number of binding sites available for metal biosorption [24]. Similar results have been reported for stem of *A. nilotica* [18] and for iron modified activated carbon [21].

Finally, this study has demonstrated the potential of iron-coated fungal biomass for the removal of As(III) at different conditions. The most attractive proposition of the biosorbent material is that it can be grown in large quantities and is cheap. The biosorbent material was successfully used for the removal of As from water samples of pole water samples having 1.0 mg/L, precedent of Zimapan, Hidalgo state, México. The mean results of water quality before and after biosorption of studied water samples are shown in Table 1. The water sample studied area is highly contaminated with As (0.6 mg/L) due to a natural contamination of the subterranean water [32–34], which indicated that it was out of the maximum allowable limit for drinking water (0.05 mg/L), according to NOM-127-SSA1-1994. Currently, it is 0.025 mg/L, according to NOM-127-SSA1-1994-2000 (1994) [35, 36]. It may be seen that after biosorption of As(III), this was reduced to a value of 0.270 mg/L, showing the efficiency of biosorbent material for the removal of As(III) ions from pole water samples, and the results are similar for arsenical removal with stem of *A. nilotica*, Ionic Exchange Resins, and Maracuyá [18, 37, 38].

## 4. Conclusion

In this study, As(III) uptake by iron-coated fungal biomass was investigated. The performance of the biosorbents was examined as a function of the operating conditions, in particular incubation time, pH and initial metal ion concentration, and fungal biomass. The experimental evidence shows a strong effect of the experimental conditions. Maximum biosorption capacity values showed that the modified biosorbent used is very effective in recovery or removal of As(III) ions from aquatic systems. When the ease of production and economical parameters is concerned, it was observed that *Paecilomyces *sp. is a very promising biomaterial for removal or recovery of the metal ion studied.

## Figures and Tables

**Table 1:** Removal of As(III) of natural water contaminated with 1.0 mg/L of As(III), 5 g of iron-coated biomass, 100 rpm, 30°C, pH 6.5 (adjusted), 24 h of incubation.

As(III) concentration	Before biosorption	After biosorption	Removal
(mg/L)	1.0 (100%)	0.270 (27%)	0.730 (73%)
